# 单倍体造血干细胞移植治疗伴重现性遗传学异常进展期骨髓增生异常综合征14例临床分析

**DOI:** 10.3760/cma.j.cn121090-20251113-00525

**Published:** 2026-01

**Authors:** 海涛 高, 菲菲 唐, 圆圆 张, 翼飞 程, 晓冬 莫, 婷婷 韩, 萌 吕, 峰蓉 王, 晨华 闫, 于谦 孙, 育红 陈, 伟 韩, 昱 王, 兰平 许, 晓辉 张, 晓军 黄, 翔宇 赵

**Affiliations:** 北京大学人民医院，北京大学血液病研究所，国家血液系统疾病临床医学研究中心，造血干细胞移植治疗血液病北京市重点实验室，北京 100044 Peking University People's Hospital, Peking University Institute of Hematology, National Clinical Research Center for Hematologic Disease, Beijing Key Laboratory of Hematopoietic Stem Cell Transplantation, Beijing 100044, China

**Keywords:** 骨髓增生异常综合征, 重现性遗传学异常, 异基因造血干细胞移植, Myelodysplastic syndrome, Recurrent genetic abnormalities, Allogeneic hematopoietic stem cell transplantation

## Abstract

**目的:**

探讨单倍体造血干细胞移植（haplo-HSCT）治疗伴重现性遗传学异常的进展期骨髓增生异常综合征（MDS）患者的疗效。

**方法:**

回顾性队列研究。纳入2015年1月至2020年12月在北京大学人民医院血液科接受haplo-HSCT的进展期MDS患者，其中14例伴重现性遗传学异常，采用1∶4倾向评分匹配（PSM）方法选择出56例不伴重现性遗传学异常的进展期MDS患者，匹配因素包括年龄和疾病阶段［伴原始细胞增多（EB）-1或EB-2］。比较两组患者的临床结局。

**结果:**

伴、不伴重现性遗传学异常组haplo-HSCT后5年总生存率分别为（64.3±12.8）％、（66.6±6.6）％（*χ*^2^＝0.044，*P*＝0.833），无病生存率分别为（57.1±13.2）％、（59.8±6.7）％（*χ*^2^＝0.031，*P*＝0.861），非复发死亡率分别为（21.4±11.5）％、（21.4±5.5）％（*χ*^2^＝0.004，*P*＝0.952），累积复发率分别为（21.4±11.5）％、（16.1±5.0）％（*χ*^2^＝0.096，*P*＝0.757）。

**结论:**

伴、不伴重现性遗传学异常MDS患者haplo-HSCT治疗后预后无明显差别。Haplo-HSCT是伴重现性遗传学异常的进展期MDS患者的可行选择。

## Abstract (en)

**Objective:**

To evaluate the efficacy of haploidentical hematopoietic stem cell transplantation（haplo-HSCT）in advanced myelodysplastic syndrome（MDS）patients with recurrent genetic abnormalities.

**Methods:**

We conducted a retrospective analysis of the clinical characteristics and prognosis of 14 advanced MDS patients with recurrent genetic abnormalities who underwent haplo-HSCT in the Hematology Department of Peking University People's Hospital from January 2015 to December 2020. The present study employed a 1∶4 propensity score matching method to select 56 contemporaneous advanced MDS patients without recurrent genetic abnormalities as the control group. The matching factors included age and disease stage（EB-1 or EB-2）.

**Results:**

The 5-year overall survival and disease-free survival after haplo-HSCT for MDS patients with and without recurrent genetic abnormalities were（64.3±12.8）％ *vs*（66.6±6.6）％（*χ*^2^＝0.044, *P*＝0.833）and（57.1±13.2）％ *vs*（59.8±6.7）％（*χ*^2^＝0.031, *P*＝0.861）respectively. The 5-year cumulative non-relapse mortality rates after haplo-HSCT for MDS patients with and without recurrent genetic abnormalities were（21.4±11.5）％ and（21.4±5.5）％（*χ*^2^＝0.004, *P*＝0.952）, respectively; the relapse rates were（21.4±11.5）％ and（16.1±5.0）％（*χ*^2^＝0.096, *P*＝0.757）, respectively.

**Conclusion:**

These results suggest that the prognosis of MDS patients with recurrent genetic abnormalities is comparable to that of MDS patients without these abnormalities after haplo-HSCT. Haplo-HSCT may be an effective therapeutic option for advanced MDS patients with recurrent genetic abnormalities.

在既往的WHO分类中，骨髓增生异常综合征（MDS）和急性髓系白血病（AML）之间的原始细胞阈值界定为20％[1]–[2]。第5版WHO造血与淋巴组织肿瘤分类取消了对具有重现性遗传学异常AML患者20％原始细胞的要求[3]。根据新的国际共识分类（ICC）和欧洲白血病网（ELN）分类[4]，诊断为具有重现性遗传学异常的AML仍需要外周血或骨髓中至少10％的原始细胞。对于既往诊断为MDS（原始细胞<20％）且伴有AML相关重现性遗传学异常的患者，按照新的WHO标准应该被诊断为AML，我们将这部分患者（原始细胞<20％）称为伴重现性遗传学异常的MDS。根据新的WHO标准，除AML伴RUNX1::RUNX1T1、CBFB::MYH11、PML::RARA、NPM1和CEBPA突变外，其他伴重现性遗传学异常的AML（AML伴DEK::NUP214融合、MECOM重排、KMT2A重排等）属于预后不良组。

伴重现性遗传学异常的MDS相对少见，只有少数小样本报道[5]–[6]。MDS的唯一根治性疗法是异基因造血干细胞移植（allo-HSCT）[7]–[8]。对于高危MDS患者，allo-HSCT已显示出较非移植疗法更好的治疗结果[9]–[10]。对于伴重现性遗传学异常的MDS患者，目前还没有专门探讨allo-HSCT作用和价值的报道。我们对接受单倍体造血干细胞移植（haplo-HSCT）的进展期MDS患者［MDS伴原始细胞增多（MDS-EB）-1或EB-2］进行回顾性分析，旨在评估haplo-HSCT对伴重现性遗传学异常进展期MDS患者的疗效。

## 病例与方法

一、病例

本研究为回顾性队列研究，纳入2015年1月至2020年12月在北京大学血液病研究所接受haplo-HSCT的进展期MDS患者。排除标准：①MDS患者移植前的任何时间骨髓或外周血原始细胞≥20％；②伴有RUNX1::RUNX1T1、CBFB::MYH11和PML::RARA融合基因的患者。最终入组了14例伴重现性遗传学异常的MDS患者，包括DEK::NUP214融合、MECOM重排、KMT2A重排和NUP98重排4个重现性异常。对于每例重现性遗传学异常的MDS患者，采用1∶4倾向评分匹配（PSM）方法选择对照，匹配因素包括年龄和疾病阶段（EB-1或EB-2）。最终，56例不伴重现性遗传学异常的MDS患者纳入本研究。MDS的诊断和分类标准参考2016年WHO标准。本研究已获得所有患者或其监护人的知情同意，根据《赫尔辛基宣言》进行。

二、预处理及移植物抗宿主病（GVHD）预防

预处理：阿糖胞苷（Ara-C）4 g·m^−2^·d^−1^，−10 d、−9 d；白消安3.2 mg·kg^−1^·d^−1^，−8～−6 d；环磷酰胺1.8 g·m^−2^·d^−1^，−5 d、−4 d；司莫司汀250 mg/m^2^，−3 d；兔抗人胸腺细胞球蛋白2.5 mg·kg^−1^·d^−1^，−5～−2 d。所有患者均接受环孢素A、霉酚酸酯和短程甲氨蝶呤进行GVHD预防。

三、定义

根据国际预后评分系统（IPSS）[11]和修订后的IPSS（IPSS-R）[12]在诊断时进行风险分层。根据MDS综合细胞遗传学评分系统进行细胞遗传学分层。粒细胞植入定义为中性粒细胞绝对计数（ANC）≥0.5×10^9^/L连续3 d，血小板植入定义为PLT≥20×10^9^/L连续7 d且脱离血小板输注。急性GVHD（aGVHD）和慢性GVHD（cGVHD）诊断与分级参照文献[13]–[14]。复发定义为外周血、骨髓或髓外出现形态学复发证据。非复发死亡（NRM）定义为在移植后28 d内因任何原因死亡或28 d后没有疾病复发证据的死亡。

四、随访

随访资料通过电话随访和查阅门诊/住院病历获取。末次随访时间为2023年12月31日。总生存（OS）期定义为从移植到死亡（任何原因）或末次随访的时间，无病生存（DFS）期定义为从移植至疾病复发或任何原因导致死亡或末次随访的时间。

五、统计学处理

采用SPSS 26.0和R 4.1.2进行统计学处理。使用Mann-Whitney *U*检验进行连续变量比较，使用卡方检验或Fisher精确检验进行分类变量比较。生存分析采用Kaplan-Meier方法。累积复发率（CIR）和NRM采用竞争风险分析计算，使用Gray检验检查组间差异。MDS亚型（伴或不伴重现性遗传学异常）被纳入Cox比例风险模型中。以下具有临床意义的变量被纳入多因素分析：MDS分期（EB-1/EB-2）、移植年龄（<40岁/≥40岁）、重现性遗传学异常（不伴/伴）以及移植前降细胞治疗（否/是）。*P*<0.05为差异有统计学意义。

## 结果

一、患者特征

在伴重现性遗传学异常的MDS患者中，检出以下4种重现性异常：MECOM重排（9例）、KMT2A重排（2例）、NUP98重排（2例）和DEK::NUP214融合（1例）。伴、不伴重现性遗传学异常组诊断到haplo-HSCT的中位时间分别为5.4（1.2～38.5）、7.0（2.0～73.4）个月（*P*＝0.130），中位移植年龄分别为39（7～52）、41（8～52）岁（*P*＝0.187）。伴、不伴重现性遗传学异常MDS患者峰值骨髓原始细胞比例中位数分别为13％（5％～19％）、10％（5％～19％）（*P*＝0.254），而移植时骨髓原始细胞比例中位数分别为5％（0％～14％）、7％（1％～17％）（*P*＝0.210）。伴、不伴重现性遗传学异常MDS患者中，接受移植前降细胞治疗的患者占比分别为57.1％（8/14）、37.5％（21/56）（*P*＝0.182）。另外，两组MDS患者在预后风险分层方面差异无统计学意义。患者特征详见表1。

**表1:** 伴、不伴重现性遗传学异常的骨髓增生异常综合征患者的临床特征

临床特征	伴重现性遗传学异常 （14例）	不伴重现性遗传学异常 （56例）	统计量	*P*值
移植时中位年龄［岁，*M*（范围）］	39（7～52）	41（8～52）	−1.320	0.187
患者性别［例（％）］			0.136	0.713
男	8（57.1）	35（62.5）		
女	6（42.9）	21（37.5）		
疾病分期［例（％）］			0.134	0.714
MDS-EB-1	5（35.7）	23（41.1）		
MDS-EB-2	9（64.3）	33（58.9）		
IPSS预后分组［例（％）］			0.563	0.755
中危-1	0（0）	2（3.6）		
中危-2	9（64.3）	33（58.9）		
高危	5（35.7）	21（37.5）		
IPSS-R预后分组［例（％）］			0.284	0.867
中危	0（0）	1（1.8）		
高危	3（21.4）	13（23.2）		
极高危	11（78.6）	42（75.0）		
峰值骨髓原始细胞比例［％，*M*（范围）］	13（5～19）	10（5～19）	1.141	0.254
移植时骨髓原始细胞比例［％，*M*（范围）］	5（0～14）	7（1～17）	1.210	0.210
移植前降细胞治疗［例（％）］			1.781	0.182
是	8（57.1）	21（37.5）		
否	6（42.9）	35（62.5）		
诊断至移植时间［月，*M*（范围）］	5.4（1.2～38.5）	7.0（2.0～73.4）	−1.513	0.130
供者性别［例（％）］				1.000
男	11（78.6）	44（78.6）		
女	3（21.4）	12（21.4）		
MNC输注量［×10^8^/kg，*M*（范围）］	8.8（7.1～13.7）	8.4（6.6～12.7）	1.424	0.154
CD34^+^细胞输注量［×10^6^/kg，*M*（范围）］	2.0（1.0～3.8）	2.4（0.9～6.9）	1.183	0.200
随访时间［月，*M*（范围）］	50（4～95）	55（1～109）	−0.396	0.692

**注** MDS-EB：骨髓增生异常综合征伴原始细胞增多；IPSS：国际预后积分系统；IPPS-R：修订版国际预后积分系统；MNC：单个核细胞

二、植入和GVHD

所有患者均实现中性粒细胞植活，中位植活时间为13 d。8例患者血小板未植活。伴、不伴重现性遗传学异常MDS患者血小板植入率分别为92.9％（13/14）、87.5％（49/56）（*χ*^2^＝0.067，*P*＝0.573），中位血小板植入时间分别为15（7～90）d、16（9～120）d（*z*＝0.156，*P*＝0.876）；中位中性粒细胞植活时间分别为13.5（11～21）d、12.5（10～24）d（*z*＝0.477，*P*＝0.633）。移植后1个月的供者嵌合率为100％。

至移植后100 d，伴、不伴重现性遗传学异常MDS患者Ⅱ～Ⅳ度aGVHD累积发生率分别为（42.8±9.5）％、（37.5±8.7）％（*χ*^2^＝0.034，*P*＝0.433）。14例伴重现性遗传学异常、53例不伴重现性遗传学异常的MDS患者移植后存活超过100 d，符合cGVHD评估条件。伴、不伴重现性遗传学异常组移植后2年cGVHD的累积发生率分别为（42.8±10.1）％、（46.5±10.7）％（*χ*^2^＝0.042，*P*＝0.412）。

三、复发

伴、不伴重现性遗传学异常组分别有3例、10例患者移植后出现复发，中位复发时间为移植后14（2～67）个月，移植后5年累积复发率分别为（21.4±11.5）％、（16.1±5.0）％（*χ*^2^＝0.096，*P*＝0.757）。多因素分析显示，复发与重现性遗传学异常（不伴/伴）、移植年龄（<40岁/≥40岁）、MDS分期（EB-1/EB-2）和移植前降细胞治疗（否/是）无相关性（表2）。

**表2:** 影响进展期骨髓增生异常综合征（MDS）患者单倍体造血干细胞移植预后的多因素分析

变量	*HR*	95％*CI*	*P*值
复发			
重现性遗传学异常（不伴/伴）	1.397	0.370～5.281	0.622
移植时年龄（<40岁/≥40岁）	2.482	0.733～8.413	0.144
MDS分期（EB-1/EB-2）	0.725	0.231～2.277	0.582
移植前降细胞治疗（否/是）	1.910	0.605～6.023	0.270
NRM			
重现性遗传学异常（不伴/伴）	1.228	0.342～4.412	0.753
移植时年龄（<40岁/≥40岁）	3.580	0.997～12.853	0.051
MDS分期（EB-1/EB-2）	1.854	0.606～5.670	0.279
移植前降细胞治疗（否/是）	0.608	0.198～1.862	0.383
OS			
重现性遗传学异常（不伴/伴）	1.298	0.470～3.583	0.615
移植时年龄（<40岁/≥40岁）	3.233	1.189～8.790	0.021
MDS分期（EB-1/EB-2）	1.323	0.538～3.248	0.542
移植前降细胞治疗（否/是）	0.886	0.374～2.103	0.785
DFS			
重现性遗传学异常（不伴/伴）	1.284	0.506～3.257	0.599
移植时年龄（<40岁/≥40岁）	2.823	1.184～6.734	0.019
MDS分期（EB-1/EB-2）	1.008	0.454～2.335	0.985
移植前降细胞治疗（否/是）	1.111	0.512～2.411	0.790

**注** NRM：非复发死亡；MDS-EB：骨髓增生异常综合征伴原始细胞增多；OS：总生存；DFS：无病生存

四、非复发死亡

伴、不伴重现性遗传学异常组有3例、12例患者在移植后死于NRM，中位死亡时间为4.2（0.8～13.6）个月。主要死因包括感染、aGVHD和植入功能不良。伴、不伴重现性遗传学异常组移植后5年NRM分别为（21.4±11.5）％、（21.4±5.5）％（*χ*^2^＝0.004，*P*＝0.952）。多因素分析显示，NRM与重现性遗传学异常（不伴/伴）、移植年龄（<40岁/≥40岁）、MDS分期（EB-1/EB-2）和移植前降细胞治疗（否/是）无相关性（表2）。

五、生存

伴、不伴重现性遗传学异常组移植后5年OS率分别为（64.3±12.8）％、（66.6±6.6）％（*χ*^2^＝0.044，*P*＝0.833，图1A）；DFS率分别为（57.1±13.2）％、（59.8±6.7）％（*χ*^2^＝0.031，*P*＝0.861，图1B）。在14例伴重现性遗传学异常MDS患者中，8例在移植前接受降细胞治疗，6例未接受降细胞治疗，移植后5年OS率分别为（75.0±15.3）％、（50.0±20.4）％（*χ*^2^＝0.522，*P*＝0.470），DFS率分别为（62.5±17.1）％、（50.0±20.4）％（*χ*^2^＝0.147，*P*＝0.702）。多因素分析显示，OS和DFS与移植时患者年龄（<40岁/≥40岁）相关，与重现性遗传学异常（不伴/伴）、MDS分期（EB-1/EB-2）、移植前降细胞治疗（否/是）无相关性（表2）。

**图1:**
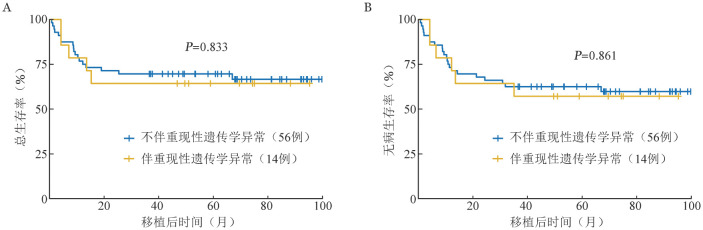
伴、不伴重现性遗传学异常骨髓增生异常综合征患者单倍体造血干细胞移植后总生存曲线（A）和无病生存曲线（B）

## 讨论

本研究结果表明，haplo-HSCT是治疗伴重现性遗传学异常MDS的有效方法，且移植后5年OS率与不伴重现性遗传学异常MDS患者的生存率相当，表明haplo-HSCT可能在一定程度上克服此类患者的不良预后。本研究中，MDS患者haplo-HSCT后的生存率与我们之前的研究[10],[15]相似，并且明显优于其他研究[16]–[17]。本研究中纳入MECOM重排、KMT2A重排、NUP98重排和DEK::NUP214融合4种重现性遗传学异常，根据2022年ELN风险分层，这4种遗传学异常通常代表预后不良[4]。既往研究表明，接受HSCT的MECOM重排MDS患者比单独接受化疗或支持治疗的患者具有相对更好的OS（分别为15.0、9.0、3.2个月，*P*＝0.029）[5]。在本研究中，共有9例MECOM重排的MDS患者以haplo-HSCT作为一线治疗，移植后中位OS期为46个月，优于上述研究。目前尚无关于haplo-HSCT治疗伴KMT2A重排和NUP98重排MDS患者的报道。Fang等[6]报道，具有DEK::NUP214融合基因MDS患者的预后较差，与具有该融合基因的AML患者相当，而allo-HSCT可以明显提高这类患者生存率。在本研究中，伴重现性遗传学异常MDS患者的移植后5年OS、DFS率分别为64.3％、57.1％，优于以前研究中的数据，可能与以下因素有关：①本研究中伴重现性遗传学异常MDS患者的中位年龄为39岁，低于其他研究中的患者年龄。②本组患者移植模式均为haplo-HSCT，而haplo-HSCT可能具有更强的移植物抗白血病效应[9],[18]。

对于进展期MDS患者，移植前降细胞治疗的作用仍不明确。对于高危AML，通常认为有必要进行移植前诱导治疗[19]。本研究结果显示，移植前降细胞治疗没有对伴重现性遗传学异常MDS患者的移植后远期生存产生有益影响，这与我们之前的研究[20]和其他研究[21]–[22]的结果相符。

综上所述，本研究结果表明，伴、不伴重现性遗传学异常的MDS患者haplo-HSCT后的预后相当。Haplo-HSCT很可能是伴重现性遗传学异常的进展期MDS患者的可行选择。本研究为单中心、回顾性研究且病例数有限，以上结论尚需多中心和前瞻性研究进行验证。
